# Empirical Bayes Methods, Evidentialism, and the Inferential Roles They Play

**DOI:** 10.3390/e26100859

**Published:** 2024-10-12

**Authors:** Samidha Shetty, Gordon Brittan, Prasanta S. Bandyopadhyay

**Affiliations:** 1 Department of Mathematical Sciences, Montana State University, Bozeman, MT 59717, USA; samidha.shetty@montana.edu; 2 Department of History and Philosophy, Montana State University, Bozeman, MT 59717, USA

**Keywords:** James-Stein estimator, Beta-Binomial distribution, confirmation/evidence distinction, Subjective Bayesianism, Objective Bayesianism, base-rate fallacy, Evidentialism, paradox of the ideal evidence

## Abstract

Empirical Bayes-based Methods (*EBM*) is an increasingly popular form of Objective Bayesianism (*OB*). It is identified in particular with the statistician Bradley Efron. The main aims of this paper are, first, to describe and illustrate its main features and, second, to locate its role by comparing it with two other statistical paradigms, Subjective Bayesianism (*SB*) and Evidentialism*. EBM*’s main formal features are illustrated in some detail by schematic examples. The comparison between what Efron calls their underlying “philosophies” is by way of a distinction made between confirmation and evidence. Although this distinction is sometimes made in the statistical literature, it is relatively rare and never to the same point as here. That is, the distinction is invariably spelled out intra- and not inter-paradigmatically solely in terms of one or the other accounts. The distinction made in this paper between confirmation and evidence is illustrated by two well-known statistical paradoxes: the base-rate fallacy and Popper’s paradox of ideal evidence. The general conclusion reached is that each of the paradigms has a basic role to play and all are required by an adequate account of statistical inference from a technically informed and fine-grained philosophical perspective.

## 1. Introduction

Although Edmond Halley (1693) constructed a table of real-life mortality figures and showed how to use it to calculate the purchase price of a life annuity, thereby laying a rigorous foundation for actuarial statistics, and Jacob Bernoulli in his *Ars Conjectandi* (1713) proved among other important results a theorem to the effect that, the more times you repeat an experiment, the likelier its results will converge, a more generalizable and equally rigorous account of statistical inference can plausibly be said to date from the publication of the Bayes Theorem in 1763. Augmented over the centuries by mathematical and, more recently, computer science and machine-learning developments, and by a finer-grained philosophical analysis, it provided the basis for what is perhaps now the most vibrant statistical inferential methodology [1]. Given that scientific practice is increasingly informed by the statistical analysis of ever-larger datasets, “Bayesianism” has become *a*, if not *the,* reigning model of scientific inference.

The focus of this paper is on the most prominent current Bayesian variant: Empirical Bayes Methods (*EBM*). A great deal of good work applying *EBM* to real-world data-driven examples has already been carried out. Ref. [1], by its foremost analyst and advocate, Brad Efron, provides a useful sampling of them. Our aim is not to comment on or add to these examples, however, but to place them in a larger more philosophical perspective, where the question “*why* is this statistical methodology important?” rather than “*how* does it apply?” is at stake. In other words, the task here is to compare what Efron calls the “philosophies” underlying the various methodologies to determine the role which each is best suited to play and to provide schematic rather than real-world data-driven examples of their applications. We want to explore foundational issues that are, for the most part, downplayed in the statistical methods literature.

There are at least five ways in which the novelty of the paper can be made clearer. First, we have been working on a crucial distinction between confirmation and evidence for more than 20 years (see [2] for the earliest, [3] for the most recent, and [4] for the most developed), spelling out its importance to the philosophical community. Our goal here is to reach out to a larger community of philosophically minded statisticians, statistically minded philosophers, and scientists whose work depends on the application of statistical methods. Second, the focus of this special issue of *Entropy* is on “evidence”; the confirmation/evidence has a special role to play. To the best of our knowledge, we are the first commentators to address the bearing of this distinction on our understanding and application of *EBM.* Third, the well-known paradox of ideal evidence was introduced by Karl Popper as a serious problem for Subjective Bayesianism (*SB*). We introduce novel ways in which Subjective Bayesians, and, for that matter, Empirically based Bayesians and Evidentialists, can deal with the Paradox. Fourth, the base-rate fallacy has been made much of for more than 40 years. In [2], we were (again, to the best of our knowledge) the first analysts to explain why this fallacy is so often committed as a result of blurring the confirmation/evidence distinction. The important novelty of its discussion here is in extending our earlier analysis to show how Evidentialists can provide a formal account of the fallacy as powerful as and consistent with that of the Subjective Bayesians. The fifth novelty is to contrast how three basic statistical methodological approaches—Subjective Bayesian, Empirically based Bayesian, and Evidentialist—handle pooling data to provide what Efron calls “indirect evidence”.

Now, we look at cases. We begin with what might be called “Basic Bayesianism”. In Section 2, the confirmation/evidence distinction is characterized and illustrated. Section 3 has to do with “Objective Bayesianism” and focuses on its most prominent “Empirically Based Methods” variant and the way in which it might seem to accommodate the confirmation/evidence distinction. Section 4 provides a comparison between “Basic” (or, given its agent-dependent interpretation of probability, “Subjective”) Bayesianism, Empirical Methods Bayesianism (*EMB*), and a third, non-Bayesian, statistical paradigm, “Evidentialism”, by way of their resolutions of two well-known inferential problems: the base-rate fallacy and the paradox of ideal evidence. Finally, in Section 5, some conclusions are drawn with respect to the three paradigms considered—principally, that each plays an irreducible role when scientific inference is analyzed from a fine-grained philosophical perspective.

## 2. Basic Bayesianism

For present purposes, *Basic Bayesianism* (*BB*) has three components. The first two are more significant in what follows:

I.   A theoretical perspective that accords special importance to the Bayes Theorem in the confirmation and acceptance of hypotheses;
II.  A particular interpretation of the Theorem;
III. A description of how rational agents both do and should readjust their beliefs on the basis of accumulating data.

The Bayes Theorem is easily derived from the axioms of probability theory and a definition of “conditional probability.” It asserts that the posterior probability of a hypothesis conditional on data, Pr(*H*│*D*), is equal to the prior probability of the hypothesis, Pr(*H*), multiplied by the likelihood of the data on the hypothesis [3], Pr(*D*│*H*), and divided by the expectedness or marginal probability of the data, Pr(*D*). In its simplest form,

**Pr(*H*│*D*) = [Pr(*H*) × Pr(*D*│*H*)]/Pr(*D*).**

*BB* is, first, the view that confirmation is a function of posterior probabilities; to confirm a theory is to raise its posterior probability, viz.,

***D* confirm H to a degree just in case Pr(*H*│*D*) > Pr(*H*).**

But, second, *BB* is an interpretation of the probability operators in the Bayes Theorem. The prior probability of a hypothesis is taken as a measure of the *degree of belief* of a rational agent in the hypothesis prior to inaugurating tests of it. As with truth in a disjunctive proposition, it spreads through the Theorem; i.e., the remaining probability operators are also given a partial belief interpretation.

There are at least two reasons for taking probabilities of hypotheses as measures of belief. One has to do with the contrast between what we *know* and what we merely *believe* to one degree or another to be the case. On it, probability is a measure of *uncertainty*, itself a function of our *ignorance* of what *is in fact* the case. The other has to do with the traditional close connection between belief and action; viz., if we want something *X* and believe that doing *Y* will help us obtain it, then other things being equal we will do *Y.* On this line of thought, probability is a measure of our willingness to bet on courses of available actions [5]. On both lines of thought, probability has to do with the epistemically uncertain situation in which we find ourselves, and, since this varies from one individual to the next, it is inevitably subjective [6].

It follows from what has been said, albeit very briefly, that *BB* neglects four potentially relevant distinctions.

**Key Distinctions**

I.   Between *confirmation* and *evidence*. In this, it finds support in ordinary language, for the words are often used interchangeably: “evidence” for a hypothesis is whatever “confirms” it, and whatever confirms a hypothesis is evidence for it.
II.  Correlatively, between *evidence* and *data.* Again, the two terms are commonly inter-defined. “Evidence” for a hypothesis is taken to consist of “data” in support of one hypothesis. Data supporting a hypothesis, in turn, provide evidence for it.
III. Between *subjective* and *objective* and interpretations of the probability operators in the Bayes Theorem.
IV. By the same token, a distinction between subjective *private* probability assignments which differ from one agent to the next and inform individual action and consensual *public* probability assignments which inform social action.

The third distinction has to be understood more carefully. *BB* recognizes that the likelihood of the data on hypotheses is agent-independent and, in this sense, objective, but the prior and posterior probabilities and the marginal probability of the data in the Theorem express degrees of belief that the given hypotheses are true or not, and are, thus, agent-relative. Since, on the *BB* account, confirmation is defined in terms of a relation between these probabilities, it too is subjective in character.

Although *Bayesianism* is rapidly becoming the dominant statistical inference paradigm, its avowedly subjective character faces the fact that *science* is, at least, in principle, *objective,* as are, in aim, the social policies which it informs. This has led some Bayesians, but certainly not all ([7,8,9,10,11,12,13] are notable exceptions), to modify *BB* in the direction of greater objectivity by placing additional constraints on allowable priors. A main aim in this essay is to describe, criticize, and eventually locate, with respect to the proper uses to which it can be put, one of its most currently popular variations, the so-called *Empirical Bayes Methods* (*EBM*). This requires first making a case for a distinction between confirmation and evidence and indicating how this might be carried out.

## 3. Why and How Should Confirmation and Evidence Be Distinguished?

In 1997, Richard Royall [14] drew attention to an intuitive distinction between three questions one could pose concerning a positive test of a particular hypothesis:

I.   Given the datum, what should we *believe* and to what degree? Or, given the datum, how should we *change* our degree of belief?
II.  Does the datum provide *evidence* for a hypothesis *H*_1_ and an alternative *H*_2_? Or, if it does, then how *strong* is this evidence?
III. Given a positive test result, what, if anything, should one *do*?

These are intuitively different questions requiring different answers. We might mark the difference in an equally intuitive way by labeling the first the *confirmation question*, the second the *evidence question*, and the third the *decision question.* The first two will occupy us in the third and fourth sections.

We *could* and we *might* pose these questions and label their answers. Doing so would find some support in ordinary language, but so too does running “confirmation” and “evidence” together. More to the point, running them together has paradoxical consequences. Does distinguishing them resolve these consequences? A commonplace example argues that it does.

Suppose that a patient complained of a persistent cough to her doctor, who then recommended that she have a chest X-ray. Assume that the X-ray, a routine test for tuberculosis (TB), comes back positive. On the basis of a very extensive sample, viz., 100,000 people, the probability of a positive X-ray for those people infected with TB is near 0.7333 and the probability of a positive X-ray for those people not similarly infected is near 0.0285. Denote this background information regarding such probabilities as *B.* Let *H*_1_ represent the hypothesis that an individual tested is suffering from tuberculosis and ~*H*_1_ that she is not. These two hypotheses are clearly mutually exclusive and jointly exhaustive. Finally, assume that *D* represents a positive X-ray test result.

The task is to find Pr(*H*_1_│*D & B*), the posterior probability that an individual who tests positive for tuberculosis actually has the disease. The Bayes Theorem enables us to obtain that probability. In order to apply the Theorem, however, we first need to know Pr(*H*_1_), Pr(~*H*_1_), Pr(*D*), Pr(*H*_1_ *& B*), and Pr(*D*)│(~*H*_1_ *& B*). Pr(*H*_1_) is the prior probability that an individual in the general population has tuberculosis. Because the individuals in different studies who showed up in the medical records were not chosen from the population at random, the correct frequency-based prior probability of the hypothesis could not be inferred from the large dataset referred to above. Yet, in a 1987 survey [15], there were 9.3 cases of tuberculosis per 100,000 people. Consequently, Pr(*H*_1_) = 0.000093. Hence, Pr(~*H*_1_) = 0.999907. As already indicated, we may take the following probabilities at face value: Pr(*D│*(*H*_1_ & *B*), the probability of a positive X-ray given that an individual has tuberculosis, = 0.7333; and Prob(*D│~H*_1_ & *B*), the probability of a positive X-ray given that a person does not have tuberculosis, = 1 − Pr(~*D│~H*_1_ & *B*) = 1 − 0.9715 = 0.0285. Using all of this information, we compute Pr(*H*_1_*│D* & *B*), the probability that an individual has TB given a positive test result and available background information, as 0.00293.

Because most people do not have tuberculosis, this seemingly paradoxical conclusion follows from the fact that, although the test has fairly good specificity, most positive tests are false positives. For every 100,000 positive X-rays, only 239 signal genuine cases of tuberculosis. It follows that the posterior probability of tuberculosis given a positive test result is very low, although it is slightly higher than the prior probability.

How is this seemingly paradoxical conclusion to be resolved? It is resolved by utilizing the distinction already drawn between confirmation and evidence. The difference between posterior and prior probabilities, our measure of confirmation, is only 0.00284. The hypothesis that the patient tested has TB is not very well-confirmed. If, however, we characterize evidence in terms of a likelihood ratio; viz.,

*D* is *evidence* for *H*_1_ *& B* as against *H*_2_ & B just in case the ratio of their likelihoods, LR is greater than 1 [16]; i.e.,

**Pr(*D│H*_1_
*& B*)/Pr(*D│H*_2_ & *B*) > 1**

The paradox disappears. Since the LR, 0.7333/0.0258, is very high, viz., 25.7, a positive test result for TB has a great deal of evidential significance. Briefly put, the seemingly paradoxical conclusion results from a confusion between evidence and confirmation. Even though these terms are used interchangeably in ordinary language, they should be distinguished sharply [17].

In fact, our way of distinguishing evidence and confirmation (for other examples, see [18,19]) has at least three corollaries.

**Corollaries of the Distinction Between Evidence and Confirmation**

I.  Data constitute evidence only in so far as they manage to discriminate between hypotheses; i.e., data constitute evidence only within a comparative context when two or more hypotheses are being evaluated. Data confirm hypotheses singly, one at a time, and not necessarily in a comparative context.
II. Confirmation is agent-dependent and, in this sense, subjective, for it has to do with increasing or decreasing the belief that particular hypotheses are true. Evidence is agent-independent and, in this sense, objective, since it has to do, on the one hand, with the logical relationship between data and models (data-generating hypotheses) and, on the other, with an arithmetical relationship between likelihoods and not with what the agent knows/believes about the data and the competing hypotheses (models) at stake.
  - As just indicated, confirmation is committed to the view that hypotheses are true or false; i.e., the determination of prior probabilities is with respect to a set of hypotheses, one of which must be true (since probability theory requires that the probabilities of the hypotheses under consideration must sum to 1).
  - It follows as well that the degree of confirmation of a particular hypothesis, the distance between prior and posterior probabilities, must range between 0 and 1.
  - Moreover, confirmation is conceptually kinematic: its measure changes over time as data accumulate, with evidence being conceptually static, a timeless relationship between competing hypotheses and a set of relevant data (understandably, as the dataset changes, so too does the evidential relationship).
  - The closer to 1, the greater the degree of confirmation. Evidence on the other hand, an arithmetical ratio, can range, in principle, between -∞ and +∞. The larger the ratio, from < 1 on up, the stronger the evidence. A ratio of 8 or more is often taken as reasonably “strong” evidence.

In the case where the two hypotheses, *H*_1_ and *H*_2_, are logically incompatible and jointly exhaustive, **LR > 1 just in case Pr(*H*_1_*│D*) > Pr(H)**, *D* provides evidence for *H* if and only if *D* confirms H. In general, however, and as our calculations of evidence and confirmation in the TB case demonstrate, there is no linear relationship between the two; i.e., the strength of evidence and degree of confirmation vary widely: weak evidence/strong confirmation, both weak/strong, or strong evidence (as above)/weak confirmation. In fact, for a set of three mutually exclusive and jointly exhaustive hypotheses, one can show that evidence is possible against an incrementally confirmed hypothesis.

## 4. Empirical Bayes Methods and the Confirmation/Evidence Distinction

As the application of Bayesian methods to the analysis of complex data and the assessment of hypotheses that rested on these data became increasingly widespread, so too did the effort of many Bayesians to modify BB to varying extents in an *objective* direction consistent with traditional claims on behalf of the objectivity-guaranteeing and normative character of the “scientific method.” De Finetti had argued a generation earlier that adequate objectivity followed from the fact that a rational agent’s beliefs had to satisfy the rules of the probability calculus (for, otherwise, on a familiar Dutch Book argument, she was sure to place continually losing bets). It follows that, no matter how far apart the prior probabilities of any two such agents are, their two posterior probabilities would eventually converge, subject to certain strong conditions [19]. An expression often used was that prior probabilities would simply “wash out” over time as more and more data accumulated.

A succeeding generation of self-described “Objective Bayesians” (*OB*s) [20,21,22] focused its attention on prior probabilities (in what follows, their position is referred to as “traditional Objective Bayesianism”). *BB*s had it that it did not matter that prior probabilities were sometimes no more than hunches. They would “wash out” in any case. OBs held that it was much better to require objective priors from the outset rather than waiting for them to wash out after an unspecified interval [23]. If they were, then agent-invariant inductive inference would be possible, a form of reasoning that, like its deductive parallel, led to “logical” conclusions that any fully rational agent should accept.

The key point was that background knowledge determines the selection of priors. As Jon Williamson put it [24] (pp. 1–12, emphasis added), “The aim of Objective Bayesianism is to constrain degrees of belief in such a way that only one value of [the prior probability] will be deemed rational on the basis of an agent’s background knowledge… objective Bayesian probability varies as background knowledge varies but *two agents* with the same background knowledge *must adopt the same* probabilities as their rational degree of belief” [24]; i.e., a scientific inference is *scientific* not so much as it is agent-or-observer-*independent,* as it is agent-or-observer-*invariant*.

*OB* as just characterized can be criticized in several different ways. The one at stake in this paper is that it provides too narrow an account of statistical scientific inference. Many scientific inferences involve weighing the evidence for and against particular hypotheses and their alternatives. But such weighing of the evidence does not involve reasoning, whether probabilistic or deductive, from data premises to a hypothesis conclusion. It is, therefore, not “logical”, as the traditional Objective Bayesian claims. Two arguments lead to this conclusion.

One argument turns on an example of Efron’s (taken from [25]). Consider four dice: D_1_, D_2_, D_3_, and D_4._ D_1_ has 4 pips on four faces and 0 on two. D_2_ has 3 pips on all six faces. D_3_ has four faces with 2 pips and two faces with 6. D_4_ has 5 pips on three faces and 1 pip on three faces. If D_1_ is rolled against D_2_, D_1_ will win; i.e., it will have a higher number two-thirds of the time. If D_2_ is rolled against D_3_, then D_2_ will win two-thirds of the time. If D_3_ is rolled against D_1_, then D_1_ wins two-thirds of the time. Hence, D_1_ beats D_2,_ which beats D_3_, which, in turn, beats D_1_, each beating the other two-thirds of the time. D_3_ beats D_4_ because, half of the time, 1 pip will turn up on D_4_, in which case D_3_ will win. The other half of the time, 5 pips will show up on D_4_, in which case D_3_ will win one-third of the time. Because D_3_ can win in these two different ways, D_3_ beats D_4_ two-thirds of the time. Likewise, D_4_ beats D_1_ two-thirds of the time. Thus, the example demonstrates the failure of transitivity and is not “logical” in a standard sense.

The other argument bolsters one of Fisher’s key claims. It has to do with the contextual character of the evidential relationship between models and data. On the one hand, evidence is contextual in character. Data provide evidence for hypotheses over one or more of their rivals by way of their relative likelihoods on these hypotheses. Because of the contextual nature of the evidential relation between data and hypotheses, strong evidential support for one hypothesis over another does not imply that we have more reason to believe that the hypothesis is true; i.e., as the TB example illustrates, the latter does not *follow from* the former; i.e., the inference is once again not “logical.” On the other hand, the ratio between likelihoods in terms of which evidence is characterized is not a probability measure. In this respect, we agree with Fisher’s dictum [26] that, “though some uncertain inferences can be rigorously expressed in terms of probability theory, it does not follow that mathematical probability is an adequate concept for the rigorous expression of *uncertain inferences of every kind.”* Of course, this argument turns on the distinction we have made between confirmation and evidence. But, as has already been indicated, in its failure to make the distinction, traditional OB is not in a position to resolve the intuitions otherwise at odds in the TB example.

We are now in a position to discuss another version of Objective Bayesianism, whose principal author and advocate is Bradley Efron. [27,28,29] provide an overview of his position. It shares many of the motives of *OB* as well as its attempt to make precise what the determination of a truly rational prior requires. This variant is often termed Empirical Bayes Methods (EBM). It is perhaps at present the most persuasive objectivist modification of BB and, of particular interest here, raises two fundamental questions: *can* a Bayesian statistician make the confirmation/evidence distinction within the *EBM* framework? If she could make it, does she *need* to do so?

Here is a final note before getting down to cases and not losing sight of the forest as we proceed to describe some of the more prominent trees. In its move to constrain prior probabilities, objectivist Bayesians standardly align them with relative frequencies. Our TB example did so. Where they sometimes part company is with respect to the ways in which these frequencies are to be estimated. Writ large, *EBM* provides technically sophisticated methods which are so powerful that they call into question the need to invoke the confirmation/evidence distinction as a non-Bayesian addition to inferential methodology. The following three sections first illustrate and explain some of the statistical techniques that Empirical Bayesians have developed over the last 50 years and then explore whether the result is able to accommodate the belief/evidence distinction or not.

### 4.1. Empirically Based Bayes Methods

One way to get a grip on EB Methods is to contrast the beta–binomial distribution with the James–Stein estimator [30,31,32]. Consider this example.

We are managing doughnut shops in Bozeman, a small town in Montana. Our intention is to know the proportion of doughnuts sold in each of our ten stores (*S*_1_, *S*_2_, *S*_3_, …*S*_10_) in November 2023. Let us assume further that the stores are positioned geographically so that sales in each store may be regarded as statistically independent of the sales in the other stores. We would like to estimate the parameter *µ*_1_ = the expected sale of doughnuts in store one in November 2023 if each month consists of 30 days, based on the sample *X*_1_ = the observed sale of doughnuts in November 2023, and, similarly, *µ*_2_ = the expected sale of doughnuts in store two in November 2023 based on the sample, *X*_2_ = the observed sale of doughnuts in November 2023, and so on for the rest of the parameters, and the observed sale of doughnuts in November 2023. The “James–Stein Phenomenon” shows that, if we are interested in estimating *µ*_1_ through *µ*_10_ simultaneously, the best guesses are not *X*_1_ through *X*_10_, respectively; rather, we obtain a better estimate for each of the parameters by a formula that makes use of all the data points than with any measure that estimates the parameters separately.

Consider taking the maximum likelihood estimate (MLE) of each population parameter based on each sample mean separately. Let *x* be the *m*-tuple of the observed values of *X*_1_, *X*_2_, …, *X_m_*, in which case *x* is the MLE estimate of μ; i.e., the *m*-tuple of population means $\mu_{1},\;\mu_{2},\ldots ,\mu_{m}.$ However, the James–Stein estimator proposes a different approach to estimate μ. It uses a shrinking factor c, calculated as follows:

$$c=\left[1-\frac{\left(m-3\right)\sigma^{2}}{\sum_{i=1}^{m}{\left(X_{i}-\bar{X}\right)}^{2}}\right]$$

Here, *m* is the number of unknown means, *σ*^2^ is the common variance of the random variables $X_{i}$, and $\sum_{i=1}^{m}{\left(X_{i}-\bar{X}\right)}^{2}$ is the sum of the squared deviations of the individual observed values $X_{i}$ from the grand average $\bar{X}.$ The James–Stein estimator for the sales of each store can be found by the following equation: $\hat{\mu_{i}}=\bar{X}$+ $c\;(X_{i}$ − $\bar{X})$. The quantity $(X_{i}$ − $\bar{X})$ is the amount by which each store’s doughnut sales differs from the sample average. This equation represents that the James–Stein estimator $\hat{\mu_{i}}$ differs from the grand average by this quantity $(X_{i}$ − $\bar{X})$ multiplied by a constant c.

The James–Stein “correction” has the effect of shifting all the values towards zero. In the James–Stein case, we obtain a better estimate of the ten parameters than what we can get in the first case, where “better” means that the *sum* of the expected squared errors is lower. In fact, James and Stein proved that the James–Stein estimator dominates the MLE estimator when *m* is three or more parameter averages, although, for any single estimate, MLE could perform better. In addition, for a specific estimate of a single parameter, the MLE and James–Stein estimates could be comparable. The fact that the James–Stein estimator dominates MLE and its cognates shows that the latter are suboptimal. The MLE and the like are not optimal in correctly estimating three or more unrelated parameters simultaneously.

We look at m=10 donut shops and the proportion of their sales that were donuts. Each shop sold a total of n=100 desserts in the month of November 2023, of which 0.40, 0.18, 0.26, 0.23, 0.31, 0.31, 0.29, 0.27, 0.14, and 0.24, respectively $\left(y_{i}\right)$, were donuts. We are interested in estimating the proportion of donuts sold at each store $(\theta_{i}).$ We let $x_{i}$ denote the number of donuts sold in each store in November 2023. Therefore, $x_{i}=n*y_{i}$.

We rewrite the James–Stein estimate and use it to estimate the $\theta_{i}$ values.

James–Stein estimator:

$$\hat{\theta_{i}}=\bar{y}+\left[1-\frac{\left(m-3\right)\sigma^{2}}{\sum_{i=1}^{m}{\left(y_{i}-\bar{y}\right)}^{2}}\right]\;\left(y_{i}-\bar{y}\right)$$

where $\sigma^{2}$ is replaced by the sample variance of $y_{i}$ $\left[\frac{1}{m}\;\sum_{i=1}^{m}{\left(y_{i}-\bar{y}\right)}^{2}\right]$ and $\bar{y}$ is the sample mean of *y_i_*

$$\bar{y}=\left[\frac{1}{m}\sum_{i=1}^{m}y_{i}\right]$$

The resulting estimates for $\theta_{i}$ for each shop are as follows:

$$\begin{array}{c} 0.3041,\;0.2381,\;0.2621,\;0.2531,\;0.2771,\;0.2771,\;0.2711,\;0.2651,\;0.2261,\;\\ and\;0.2561. \end{array}$$

Alternatively, EB-based Methods use a beta–binomial distribution to run their Bayesian inference engine. A beta–binomial distribution is a mixture of a binomial and a beta distribution. A distribution is beta–binomial if *p*, the probability of success, in a binomial distribution, has a beta distribution with shape parameters α > 0 and β > 0. The shape parameters characterize the probability of success. For large values of α and β, the distribution approaches a binomial distribution. When *α* and *β* both equal 1, the distribution equals a discrete uniform distribution from 0 to *n*. When *n* = 1, the distribution is the same as a Bernoulli distribution. The beta–binomial model provides the tools we need to study the proportion of interest. 

We assume that $x_{i}\sim \;Binomial(n,\;\theta_{i})$ and $\theta_{i}\sim \;Beta\;(\alpha ,\;\beta )$. This implies that the sale of doughnuts in each shop $\left(x_{i}\right)$ follows a binomial distribution with the same number of total sales (n) and its own proportion of donuts sold ($\theta_{i}).$ The proportions themselves come from a shared beta distribution with parameters α and β. We can then use the following relationship to estimate each $\theta_{i}$,

$$\hat{\theta_{i}}=\;\frac{x_{i}+\hat{\alpha }}{\hat{\alpha }+\hat{\beta }+n}$$

When the parameter n is the same for all the shops, the Empirical Bayes method and the method of moments yield the same results. The study of moments is useful to measure how spread out or concentrated the numbers are in a dataset around the central value, such as the mean. Formally stated, for any random variable X, the kth moment of X is defined as the expected value of $X^{k}$. We use the method of moments to estimate α and β; i.e.,

$$\hat{\alpha }=\frac{nm_{1}-m_{2}}{n\left(\frac{m_{2}}{m_{1}}-m_{1}-1\right)+m_{1}}$$

$$\hat{\beta }=\frac{\left(n-m_{1})(n-\frac{m_{2}}{m_{1}}\right)}{n\left(\frac{m_{2}}{m_{1}}-m_{1}-1\right)+m_{1}}$$

where $m_{1}=\frac{1}{m}\;\sum_{i=1}^{m}y_{i}$ is the sample estimate of the first moment and $m_{2}=\frac{1}{m}\;\sum_{i=1}^{m}y_{i}^{2}$ is the sample estimate of the second moment.

The resulting estimates for $\theta_{i}$ for each shop are as follows:

0.3450, 0.2133, 0.2612, 0.2432, 0.2911, 0.2911, 0.2792, 0.2672, 0.1893, and 0.2492.

**Figure 1:**
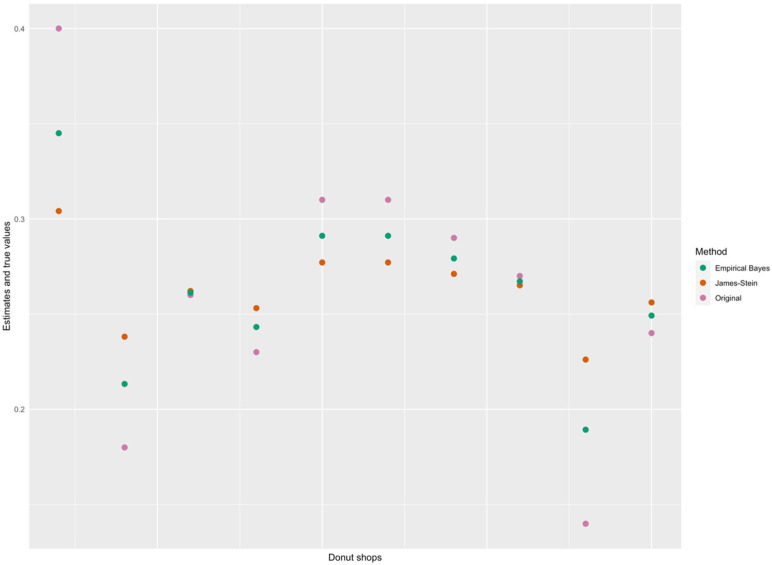
Scatterplot of the true and estimated values (using EB and James–Stein methods) of the proportion of donut sales at the 10 shops.

We can compare the values of each estimate arrived at by applying a beta–binomial distribution with the one used by applying the James–Stein’s estimate to the dataset. They are very similar to one another. For example, the James–Stein estimate provides 0.3041 for store one, whereas the EB Methods based on a beta–binomial distribution yields 0.3450 for the same store; this is also the case for the closeness of all values furnished by both the James–Stein estimate and the beta–binomial-based EB Methods. This is *not* always true but is so in our example. However, it is important to note that two methods *need not* deliver similar results in all cases. In Figure 1 below, we plot the $y_{i}\;$ values and the estimates using the EB and James–Stein methods for the 10 donut shops. Note how the estimates for both methods are similar and are shifted towards the overall mean.

Recall that we looked at m=10 donut shops and the proportion of their sales that were donuts. Each shop sold a total of n=100 desserts in the month of November 2023, of which 0.40, 0.18, 0.26, 0.23, 0.31, 0.31, 0.29, 0.27, 0.14, and 0.24, respectively $\left(y_{i}\right)$, were donuts. If we simply change the values of n=100 desserts in the month of November 2023 to 0.10, 0.18, 0.26, 0.13, 0.21, 0.31, 0.29, 0.27, 0.14, and 0.56, then the James–Stein estimates are 0.2015, 0.2225.0, 0.2495, 0.2105, 0.2345, 0.2645, 0.2585, 0.2525, 0.2135, and 0.3395. In contrast, the Empirical Bayes estimates are 0.1157861, 0.1870765, 0.2583670, 0.1425200, 0.2138104, 0.3029235, 0.2851009, 0.2672783, 0.1514313, and 0.5257061. On this new version of the donut example, we received two different sets of values from the application of the James–Stein estimates and the Empirical Bayes estimates.

**Figure 2:**
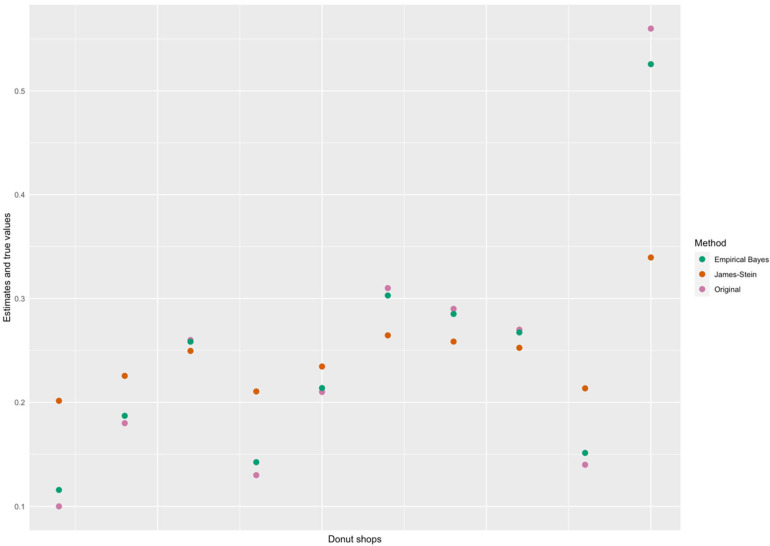
Scatterplot of the true and estimated values (using EB and James–Stein methods) of the proportion of donut sales at the 10 shops.

In Figure 2 below, we can see how the estimates for both methods are not as similar for this example as before. The estimates are, however, still shifted towards the overall mean. A possible concern could be that there is room for subjectivity even from the use of the same data. To alleviate this concern, an Empirical Bayesian could reply that there is no scope for subjectivity in the data, because the differences in the value from the application of the procedure depend entirely on the use of two distinct measures. It is in this manner that EBM both extends the applicability and underlines the power of the Bayes Rule.

So far, we have discussed how a better estimate of the prior probability of each ten-doughnut store’s sale for November for the year 2023 can be estimated using other stores’ average sales along with the help of statistical tools. There are at least two ways that an Empirical Bayesian statistician can estimate the prior distribution of each store’s sale in 2023 based on data: the grand average and the James–Stein estimate or a beta–binomial distribution.

Once we estimate the prior for each store’s sale in November 2023, we can proceed to check whether the empirically based method can compare competing hypotheses relative to data and use the Bayes theorem based on data in the manner we did in Section 2. Efron used this example to illustrate how one could do so [33]. An educationist friend of Efron, after learning that she was going to have twin boys based on sonograms, asked, “What are the chances that my twins will be identical to fraternal?” Making some further probes into the information she had, Efron came to realize that two pieces of relevant information were available regarding the question. They are as follows: (i) one-third of twins are identical and identical twins are twice as likely to provide twin boy sonograms as they are *always* of the same sex, and (ii) the likelihood of fraternal twins having the same sex is 50:50. That is, [Pr(identical)/Pr(fraternal)] = 1/2. The likelihood ratio of the current data is two-to-one in favor of identical:

$$\mathrm{Pr}[((\mathrm{same}\;\mathrm{sex})|\mathrm{identical})]/\mathrm{Pr}[(\mathrm{same}\;\mathrm{sex}|\mathrm{fraternal})]=\frac{\frac{1}{3}}{2/3}=\frac{1}{2}$$

Bayes’ theorem provides us with the answer for finding the probability of twin boys being identical given the sonogram reading of twin boys.


$$\begin{array}{c} \mathrm{Pr}(\mathrm{twins}\;\mathrm{identical}|\mathrm{sonogram}\;\mathrm{showing}\;\mathrm{twin}\;\mathrm{boys})=\\ \frac{\mathrm{Pr}(\mathrm{Sonogram}\;\mathrm{showing}\;\mathrm{twin}\;\mathrm{boys}|\mathrm{Twin}\;\mathrm{identical})\times \mathrm{Pr}(\mathrm{Twin}\;\mathrm{identical})}{\mathrm{Pr}(\mathrm{Sonogram}\;\mathrm{showing}\;\mathrm{twin}\;\mathrm{boys})}=\frac{\frac{1}{2}\times \frac{1}{3}}{1/3}=\frac{1}{2} \end{array}$$

### 4.2. Indirect Evidence: Combining Pieces of Data for a Stronger Conclusion

Efron and Hasti [4] (p. 88) write “Bayesian inference provides a theoretical basis for incorporating indirect evidence, for example, [when] the doctor’s prior experience [of a diagnosis of a disease is available]… The assertion of a prior density *g*(ⱷ) amounts to a claim for the relevance of past data to the case at hand.” They go on to say that “philosophically…, the biggest change [in the twenty-first century] has been the increased acceptance of indirect evidence, especially as seen in Empirical Bayes and Objective (“uninformative”) Bayes applications.” In this section, we will address “indirect evidence” considering Subjective Bayesianism, Evidentialism, and the EB-Methods-based Bayesianism all in one swoop.

Consider two hypotheses, “the fair coin” hypothesis, that the probability of heads equals 0.5, and “the biased coin” hypothesis, that the probability of heads equals 0.8. The first sequence of coin tosses with four heads and a tail is taken to be evidence for the biased coin hypothesis over the fair coin hypothesis. The LR of the biased/fair = 0.4096/0.1562 = 2.62 provides evidence for the biased coin hypothesis, but it is weak. The following Table 1 shows a summary of fair/biased coin flip results.

However, if the biased coin hypothesis is false, then the probability of misleading evidence is 0.1562. The probability of misleading evidence is the probability that shows up in a 2 × 2 contingency table when the hypothesis is incorrect. After receiving 4 heads out of 5 tosses, suppose we have received the following sequences of heads in each of 5 tosses by five people: 2, 3, 2, and 1 heads yielding 12 heads out of 25 tosses. After we have gathered more data, we have now received very strong evidence for the fair coin hypothesis over its biased coin alternative. LR (Fair/Biased) = 0.155/0.000298 = 529.396 times. If the fair coin hypothesis is, in fact, false, but we have strong evidence for it, then the probability of misleading positive evidence is only 0.000298, which is appreciably smaller than 0.1562 when we have weak evidence for the biased coin hypothesis. This shows that, as evidence becomes strong, the probability of misleading evidence goes down, subject to some constraints.

In the above example, we are testing two hypotheses:

**H1.**  *The coin is a fair coin; i.e., the proportion of heads is 0.5.*

**H2.**  *The coin is a biased coin; i.e., the proportion of heads is 0.8.*

The alternative form of the likelihood ratio is as follows:

*L*1 = Pr(*D*│*H*1 & *B*)*P*(*H*1)Pr(*D*│*H*2 & *B*)*P*(*H*2) = 529.396 × *P*(*H*1)*P*(*H*2) = 529.396 × 0.505 = 529.396

And we use the data generated to obtain likelihood estimates that indicate evidence in support of the hypothesis that the coin is fair. To work through a Subjective Bayesianism account of the above fair coin versus a biased coin (with a probability of heads = 0.8) example, we need to enrich the example with more details. Consider a scenario in which five people draw at random from an urn, which may contain either biased (probability of heads = 0.8 as in the previous example) or fair coins. Their first task is to flip each coin five times after drawing each of them at random from that urn, and then to infer from those data whether the urn contains an equal number of unbiased and biased coins. Since none of them knows which coin is biased or fair when each draws coins from the urn, the appropriate prior probability for each coin to be biased or fair on each draw should be 0.5. Since each draw of a coin as fair has been assumed to have a prior probability of 0.5, it follows that the prior probability of that urn having an equal number of fair and biased coins should also be 0.5. Suppose that each draws five coins at random, and, when they flip each of them one time, all end up with the results as the way we described them earlier: 4, 2, 3, 2, and 1 head(s). When the data are pooled, each person’s degree of confirmation that the urn contains an equal number of biased and fair coins has changed from a prior probability of 0.5 to a posterior probability of 0.99808. Here, indirect evidence is based on pooling both priors for the models with the data for two competing models.

As has already been indicated, the James–Stein method is a paradigm example of the use of indirect evidence. To repeat, it is an estimation technique to use multiple samples to generate estimates for each sample by centering the estimates. The formula for each parameter *θi*′*s* estimate is given by

$$\theta_{i}=\bar{x}+\left[1-\frac{\left(m-3\right)\sigma^{2}}{\sum_{i=1}^{m}{\left(x_{i}-\bar{x}\right)}^{2}}\right]\;\;(x_{i}-\bar{x})$$

where *m* is the number of samples and $x_{i}\;$ is the value of the estimate from the *i*th sample.

For the above example, $\theta_{i}$ denotes the average proportion of heads every toss, and $x_{i}$ denotes the proportion of heads in five tosses for the *i*th sample. We have m = 5 samples.

The samples generated an average proportion of 0.8, 0.4, 0.6, 0.4, and 0.2, respectively.

Using the James–Stein estimator, these above proportions can be centered, and we obtain 0.672, 0.432, 0.552, 0.432, and 0.312, respectively, for each sample.

While the likelihood method uses data to recenter the evidence towards a hypothesis about a parameter, the EB-based James–Stein estimator can be thought of as using data to recenter individual estimates of the parameter of itself. Bayesianism opens the door to both by way of the need to determine prior probabilities. Its OB and SB variants do so in different ways, however, with respect to their determination. It turns on the distinction between informative and non-informative priors. The former provide specific information about a parameter of interest, by way of the relative frequency with which it appears in a population, for instance, the latter when such information is not available or not at stake (as in the OB emphasis on formal rather than content constraints). It follows that non-informative priors have little influence on Bayesian inferences, informative priors more to one degree or another; in this respect, EBM shares elements with both. But, as should be clear from the illustration of EBM calculations in the fair coin case, the inference is objective and depends on an informative prior. Table 2 below illustrates these relationships.

By way of contrast with the subjective and EBM Bayesian variants, Evidentialism does not have a way to pool indirect evidence. As Bayesians understand the term, “evidence” consists of data confirming/disconfirming individual hypotheses and not, as on our account, of data more likely on one hypothesis than another. Moreover, on its subjective variant, the Bayesian calculation *requires* an appeal to indirect evidence. In this respect, it contrasts with traditional Frequentism. It contrasts, in another respect, with Evidentialism, for which the distinction between “direct” and “indirect” evidence does not have a role to play. Data that tip the scales in favor of one model or any of the others with which it is being compared count as “evidence.” As a further contrast with EBM, it might be noted that, while Evidential methodology, like that of most inferential paradigms, is frequency-driven, the interpretation of probability that it incorporates is not. That is, the likelihood of the data at stake is a function of the model by which they are entailed; i.e., the probability is not Frequentist but deductive/logical. Models differ from hypotheses in several ways, among which is the fact that they are invariably mathematical, but, more specifically, in that they are mechanisms generating probability distributions of data. Those models whose distributions more closely match the observed data are better supported by the evidence. This said, our earlier discussion of the base-rate fallacy demonstrates that the Evidentialist can incorporate data-driven probabilities into model comparisons when priors are derived from the data as well as EBM. This exemplifies the incorporation of indirect evidence. Moreover, it is compatible with Evidentialism to use the James–Stein estimator in providing better predictions on the basis of statistical inferences. In sum, although this account of evidence does not depend upon or even recognize a distinction between direct and indirect evidence, it can accommodate both. A merit of EBM is that it calls attention to the need to do so.

### 4.3. Revisiting the Confirmation/Evidence Distinction

We are now equipped to answer the two fundamental questions raised by EB-based Methods. The first question is as follows: *can* a Bayesian statistician make the confirmation/evidence distinction within the EBM framework? Recall that, as framed by Royall, the evidence question asks, “What do the data say about evidence for the hypothesis against its alternative and how much?” Applying it to the twin boys example, we can reformulate it as “What does the observed sonogram report (that is, there are twin boys) provide evidence for the identical twin hypothesis as against the fraternal twin hypothesis?” We know that it is two times more likely that a sonogram report provides the chance of having identical twins over fraternal twins. In contrast, the confirmation question asks, “Given the data, to what extent should an agent believe that the hypothesis that twins are identical is true?” The answer to this question is half; i.e., each is equally likely. Therefore, the EB-based Methods could be used to both weigh the evidence for/against the identical twin hypothesis and confirm it. Efron’s physicist’s friend’s worry and how to resolve it is at least similar to our analysis. We asked the following question: to what extent should an agent believe that the person in question has tuberculosis given that her X-ray is positive? The evidence question was as follows: given his X-ray is positive, what does it say about evidence that the individual in question has tuberculosis against its alternative and how strong is it? In short, it would seem that the *EB*-based statisticians *can* distinguish and reply to the two sorts of question.

But it is important to note that the *EB* statistician modifies the confirmation question in the process of answering it. Many “basic Bayesians” have added David Lewis’ *Principal Principle* to their toolkits [34]. The idea behind this principle is that, when the objective probability of a hypothesis is available, as in the TB and identical twin examples, a rational agent needs to align her belief probabilities with it. When it is not available, she is free to begin her Bayes Theorem calculation with whatever prior beliefs she has come to on the basis of whatever background information she already has. Unlike Lewis, however, the *EB*-Methods-based statisticians think that, when subjective probabilities cannot be backed by data in the ways they describe, they should be jettisoned. It follows for them that the confirmation question is no longer “given the data, what should one believe?” but “given that the data have a particular statistical character, what should one believe?” The second question we asked of statistical methodologists follows naturally on the first: if she *can* make the confirmation/evidence distinction, does she *need* to do so? Our reply to this second question will occupy us to the end of this paper. It is to the effect that our *original* distinction, resting as it did in part on a more basic distinction between belief/subjective and logical/objective probabilities, should be included in any tenable inferential paradigm.

## 5. Two Statistical Paradoxes and Their Bayesian and Evidentialist Resolutions

It helps answer our second question to compare the three statistical paradigms in play—Subjective Bayesian (classic *BB*), Evidentialist, and Empirical Bayesian (current OB)—regarding two well-known statistical problems: the base-rate fallacy made famous by Kahneman and Tversky (Section 4.1) and the paradox of ideal evidence by Karl Popper (Section 4.2).

### 5.1. The Base-Rate Fallacy

The confirmation/evidence distinction has a number of significant corollaries. One of them concerns a widespread inferential error that has been much discussed over the past generation or so. A key premise in our simple schematic demonstration that it is possible to have very strong evidence and yet a very low degree of confirmation is that, for every 100,000 positive X-rays, only 239 signal genuine cases of tuberculosis. Yet, the premise leads to a result that strikes many people as wildly counter-intuitive, that the probability of a positive X-ray for those people infected with TB is near 0.7333 and the probability that an individual has TB given that a positive test result is no more than 0.00293. It is, therefore, worth our while to examine the intuition. It involves what has come to be called “the base-rate fallacy.” In our view, the fallacy stems from the common tendency to conflate evidence and confirmation. Further, we take the fact that the base rate is so common as “evidence,” so to speak, that a distinction between them should be made.

Uncertainty is an inescapable aspect of human life. It forces us to make significant choices based on inevitably partial information. Amos Tversky and Daniel Kahneman have argued [35] that we often make mistakes when we reason probabilistically, as we must, based on such partial and uncertain information. They set out case after case of probabilistic inferential error. Perhaps the most widespread of these errors is the base-rate fallacy. It can be illustrated using a variant of the TB example just referred to. Groups of subjects are given a case and a multiple-choice question to answer with respect to it:

Someone is worried that she has tuberculosis. For a population of 100,000 positive X-rays, only 239 signal genuine cases of tuberculosis. There is a simple and effective test, which identifies the presence of tuberculosis in everyone who has it, and only gives a false positive result in 0.02 percent of the people who do not have it. She takes the test and obtains a positive result. What is now the closest probability that she, in fact, has tuberculosis?

(A) 90 percent
(B) 10 percent
(C) 50 percent
(D) 89 percent

A majority of the test-takers fail to give the correct response. Their average estimate of the probability that she suffers from the disease is 85 percent, whereas the correct answer (computed using the Bayes Theorem as we have done above) is about 10 percent. Why do they fail to understand it? According to Kahneman and Tversky, they fail because they overlook the base rate of the disease in the population; as we noted, no more than roughly 1 percent of the population suffers from the disease. In a variety of uncertain situations, we can safely ignore the base rate. This leads us, as in the Kahneman and Tversky “heuristics and biases” approach, to ignore it, generally. They explain why humans generally obtain the wrong estimate by arguing that human beings adopt certain heuristic strategies in solving problems, strategies that generally provide useful short-cuts to reasonably accurate answers, but that also bias subjects irrationally toward certain kinds of mistakes.

In our view, the distinction between confirmation and evidence provides an alternative, equally plausible, rationale for the subjects’ failure to get the TB probability right. The tendency to commit the base-rate fallacy results from the subjects’ (certainly not the investigators’) conflation of the two notions. They think the evidence question has been asked, whereas, in fact, the confirmation or belief question was asked. That is, given the data about the likelihood of positive and negative test results on the hypotheses that the people tested were and were not affected, they rightly concluded that the data provided very strong evidence that a person who tested positive was much more likely to have, rather than be free of, TB. Given the data, the evidence on our LR account of it provides close to 26 times more support for the person in question having the disease than not having it. This is *strong* evidence for the hypothesis that she has the disease, even though the confirmation of it is low. Therefore, our diagnosis of the fact that people tend, in certain kinds of cases, to ignore the base rate is that they mistakenly take the question “what is the probability post-test that she has tuberculosis?” as a question about the strength of the evidence, and not about the degree of confirmation that the data give to the hypothesis that she has the disease, which is only ten percent.

The account of evidence we have provided provides a satisfactory explanation of the base-rate fallacy if we assume certain features in the data for the competing models (someone has TB compared to someone who does not): the incorporation of the information that John Doe who has the disease has been randomly chosen from the population of 100,000 and, consequently, John Doe who does not have the disease has also been randomly chosen from the same population. These new additional data help in comparing the LR of two joint distributions of the competing models as follows. In the alternate model, we consider the following ratio:

$$\begin{array}{c} L_{1}=\frac{\mathrm{Pr}\left(D│H1\;\&\;B\right)P\left(H1\right)}{\mathrm{Pr}\left(D│H2\;\&\;B\right)P\left(\sim H1\right)}=25.729\times \frac{P\left(H1\right)}{P\left(H2\right)}\\ =25.729\times \frac{0.000093}{0.999907}\\ =25.729\times 0.000093\\ =0.002393 \end{array}$$

This differs from the previous likelihood (i.e., 25.729) by a factor of $\frac{P(H1)}{P(H2)}.$ Moreover, this is like the posterior probability for rare events, i.e., when P(H1) is small.

When H1 is rare, the posterior probability is close to the new likelihood proposed because

$$\mathrm{Pr}(H1|D)=\frac{Pr\left(D│H1\&B\right)P(H1)}{Pr\left(D│H1\&B\right)P\left(H1\right)+Pr(D│H2\&B)P(H2)}\;\approx \;\frac{Pr\left(D│H1\&B\right)P(H1)}{0+Pr(D│H2\&B)P(H2)}=L_{1}$$

The points made in this section of the paper can be summarized under three headings. First, Subjective Bayesianism, its Empirically Based Methods variant, and Evidentialism can all provide formally equivalent accounts of base-rate inclusion. Second, providing formally equivalent accounts of base-rate inclusion does not explain why the base-rate fallacy recurs so frequently in the statistical inferences people make. In identifying prior probabilities with subjective beliefs, basic Bayesianism certainly makes space for it, but none of the statistical paradigms discussed explains why the fallacy is frequent. Third, we take an explanation as tantamount to providing a general motivation. Our distinction between belief and evidence does so in a straightforward way: subjects mistakenly assume that the evidence question is being asked and, hence, provide a mistaken response to what we refer to as the confirmation question. As in the TB case, often a test that has only a very low false positive result provides strong evidence that a person is afflicted a disease, but, if the base rate of the disease in the population is low, it does not provide any more than weak confirmation that a person is afflicted. Basic Bayesians like to claim that their rule captures the way most people reason probabilistically; unfortunately, it also sometimes leads to mistaken conclusions unless supplemented by something along the lines of our belief/evidence distinction.

### 5.2. The Paradox of Ideal Evidence

Karl Popper writes, “It is a mistake to think that probability may be interpreted as a measure of our beliefs … considerations of ‘weight of evidence’ lead, within the subjective theory of probability, to paradoxes, which, in my opinion, are insoluble within the framework of that theory” [36] (p. 406). The example he instances is titled “the Paradox of Ideal Evidence.” Let *H* be the statement that “the next toss of the coin will come up heads,” where the toss is one that has not yet been observed by an epistemic agent. Popper takes the Subjective Bayesian (*SB*) to assign a probability of 0.5 to *H.* Now, assume that the same coin has been tossed a thousand times by the agent; it yields a sample of heads and tails which results in an identical assignment of 0.5 to Pr(*H│D*); i.e., despite a thousand additional flips,

Pr(*H*) = Pr(*H│D*) = 0.5.

The identity is paradoxical according to Popper because, before and after the accumulation of new data, the probability of the hypothesis remains the same; intuitively, the new data strengthen an agent’s belief that P(*H*) = 0.5, but, on a Bayesian account of confirmation, they apparently do not. This is the conundrum. Popper resolves it by dismissing the view that probability may be interpreted as a measure of our beliefs. Moreover, the “difficulties disappear as soon as we interpret our probabilities *objectively*… According to the objective interpretation, we have to introduce *b*, the statement of the conditions of the experiment” (p. 409)—in short, the relevant background information.

However, the argument that Popper develops for the claim that the prior and posterior probabilities remain the same even after the subsequent equal heads/tails data have been collected is problematic. Note, to begin with, the distance between *a* is the statement “the *n*th (as yet unobserved) toss of *z* will yield heads” (p. 407) and *h* is “the probability of tossing heads” (p. 408), i.e., between a prior belief concerning the next coin toss and a hypothesis about the unbiased character of the coin. Then, note that background information is assumed to bear on the probability of the fair coin hypothesis but not on the probability of the belief prior. As Popper puts it, “If we now consider the ideally favorable statistical evidence *e* which leads to the ’paradox of ideal evidence’, it is quite obvious from the point of view of the objective theory, *e* is considered to be the evidence bearing upon *h*, and quite neutral to *a.”* But the subjective interpretation of probability, and, with it, *BB*, does not exclude either attributing the probability of the belief prior to the fair coin hypothesis or, in doing so, bringing relevant background information informing the agent’s prior into account. As soon as we do so, the alleged paradox disappears. The probability of the first is assigned by the agent, presumably as a function of the background information available to her. The second is the probability of *H given the data gathered* as calculated on the basis of Bayes Rule. But these data raise the probability of *H* and, hence, confirm it.

Consider two mutually exclusive and jointly exhaustive hypotheses, H1 and H2. H1: fair coin (*θ* =0.5*)* and H2: biased coin (*θ ≠* 0.5 with uniform prior on all possible values), for 1000 flips where data (D) = 500 heads out of 1000 flips and Pr(D|H1) = Pr(500 heads out of 1000|θ=0.5) = 0.02523. Likewise, for

$$\begin{array}{c} Pr(D|H2)=Pr(500\;heads\;out\;of\;1000|\;\theta \;\neq \;0.5)\\ =\int_{0}^{1}\left(\begin{array}{c} 1000\\ 500 \end{array}\right)\theta^{500}{\left(1-\theta \right)}^{1000-500}\;\left(1/0.001\right)\;d\theta \;\\ =0.999.\\ Pr(H1\;|\;D)=\frac{Pr\left(D│H1\right)Pr(H1)}{Pr\left(D│H1\right)P\left(H1\right)+Pr(D│H2)Pr(H2)}\\ =(0.02523*0.999)/(0.02523*0.999+0.999*0.001)\\ =0.9619\approx 1 \end{array}$$

Hence, for 1000 flips, we find Pr(H|D) = 0.9619 > Pr(H) = ½.

For a dataset = 10,000 flips, where data = D = 5000 heads out of 10,000 flips:

$$\begin{array}{l} \mathrm{Pr}(D|H1)=\mathrm{Pr}(5000\;\mathrm{heads}\;\mathrm{out}\;\mathrm{of}\;10000|\theta =0.5\\ P(D|H2)=\mathrm{Pr}(5000\;\mathrm{heads}\;\mathrm{out}\;\mathrm{of}\;10000|\theta \;\neq \;0.5\\ =\int_{0}^{1}\left(\begin{array}{c} 10000\\ 5000 \end{array}\right)\theta^{5000}{\left(1-\theta \right)}^{10000-5000}\;\left(1/0.001\right)\;d\theta \;\\ =0.0999. \end{array}$$

$$Pr(H1\;|\;D)=\frac{Pr\left(D│H1\right)Pr(H1)}{Pr\left(D│H1\right)P\left(H1\right)+Pr(D│H2)Pr(H2)}\;\;\begin{array}{l} =(0.00798\times 0.999)/(0.00798\times 0.999+0.0999\times 0.001)\\ =0.9876=\mathrm{Pr}(H|D)>\mathrm{Pr}(H)=½. \end{array}$$

Thus, the data do confirm the original hypothesis; i.e., the Subjective Bayesian can demonstrate that the posterior probability of the hypothesis is greater than its prior.

Moreover, if the hypothesis being tested is a “fair coin” hypothesis and not that the next flip will come up heads, then the equal number of heads and tails does provide evidence, in our sense, for it as against the hypothesis that it is not a fair coin and thus provides further support for the Subjective Bayesian confirmation calculation. We have provided examples in which the confirmation and evidential results differ. Ideally, as in this case, they are mutually supportive.

In sum, the confirmation/evidence distinction helps diagnose the paradox of the ideal evidence. The diagnosis of the paradox proceeds along two lines: First, there is an ambiguity in Popper’s argument between two assertions: (i) the next toss of the coin will come up heads and (ii) the coin is fair. Each yields a different result. On the first construal, its posterior probability is 0.5, a belief probability he ascribes to Subjective Bayesianism. On the second construal, in which background information is taken into account, the posterior is 0.96, a probability that a well-informed Subjective Bayesian could derive; i.e., the diagnosis shows that, contrary to Popper, although the seeming paradox has to do with an agent’s degrees of belief, Subjective Bayesians can handle it convincingly. Second, once the paradox is seen to depend on the substitution of a “fair coin” for a “next flip” hypothesis, the allegedly paradoxical sample of 500 heads and 500 tails in a 1000 flip experiment provides an LR > 1 in support of it as against its biased alternative. An objective interpretation of confirmation is to be clearly distinguished from an objective account of evidence.

## 6. Conclusions

In his stock-taking article, “The Future of Indirect Evidence” [37], Efron has this to say about the main statistical inferential methodologies at the beginning of the 21st century:

Empirical Bayes Methods seem to me to be the most promising candidates for a combined Bayesian/Frequentist attack on large-scale data analysis problems, but they have been “promising” for 50-plus years now, and have yet to conform into a coherent theory. Most pressingly, both Frequentists and Bayesians enjoy confirmation information theories saying how well one can do in any given situation, while Empirical Bayesians still operate on an ad hoc basis (p. 158).

In our terms, *EBM* is not yet a “statistical paradigm.” Nevertheless, the identification of the ways in which it resembles and differs from the paradigms discussed in this paper—Basic/Subjective Bayesianism, classic Objective Bayesianism, and Evidentialism—is both possible and useful. That said, and as Efron emphasizes, the mass of new data and the relatively recent development of powerful mathematical and algorithmic methods to process them, call for the continuing re-examination of the statistical methods in vogue.

First, *EBM* is Bayesian in its emphasis on the Bayes Rule confirmation, its commitment to truth as the goal of statistical and, more generally, of scientific inference, and its focus on testing single and not multiple hypotheses/models. It differs from Subjective Bayesianism principally in its disallowing non-objective priors in applications of the Bayes Rule. Interestingly and in our view, it differs as well in identifying the objectivity of its methods not with the convergence of updated priors in the long run as data accumulate, but with the convergence of frequency estimations made possible by the gathering of indirect information. It differs from Objective Bayesianism in rejecting the vague requirement that two epistemic agents in possession of the same background information assign the same probability to their respective priors, and in making room for indirect as well as direct (data-gathering) information in the determination of the frequencies that, for both methodologies, should guide the choice of priors. In these large-perspective respects, *EBM* represents an attempt to find a middle ground between the Subjective and Objective camps.

Second, although it affords a way to compare competing hypotheses with respect to something like the weight of the evidence for each, EBM explicitly provides an answer to the confirmation question (in a slightly altered form), reasoning sequentially via the Bayes Theorem. In this respect, it differs rather sharply from Evidentialism, in which evidence is weighed with respect to the arithmetical relation between models whose relation to data is logical. Put simply, Evidentialism is impersonal in a more fundamental way than any Bayesian variant. Moreover, while all Bayesian variants are committed to a true hypothesis/model aim, as they must be to apply the Bayes Rule, Evidentialism is limited to weighing one model against another; while one model may be better supported by the data than another, no model is best supported by the data, since there is no upper bound to as yet unimagined models that may be better supported. By the same token, the two paradigms differ in that, while *EBM* focuses attention on models singly, Evidentialism requires that they always be evaluated in a comparative context.

We could go on in this vein. But, since the paradigms differ, the aim should not be so much to judge which one is superior but to outline the role each is best equipped to play and, in the process, to assign each its proper place in the statistician’s methodological toolkit.

Basic/Subjective Bayesianism is not well-adapted to the inference of scientifically respectable conclusions, the hallmark of which is their objectivity. Nonetheless, it plays at least two important roles. One has to do with the selection of hypotheses/models to test. Inevitably, this selection is made, at least ideally, based on an individual scientist’s beliefs that it is potentially the most explanatory, and so on, as well as testable among those she has in mind. This is where *BB’s* emphasis on the tie between belief and action comes into play. The other role it plays is with respect to unveiling the aura of paradox which surrounds, for example, the baseline fallacy and the ideal evidence paradox, for the source of the paradox is not what the rational agent would/should believe, but based on what many. if not all. people mistakenly assume is the correct prior in their informal inferences.

Objective Bayesianism is well-adapted to the inference of scientifically respectable conclusions, but its focus on the data supporting a single hypothesis and not on data differentiating multiple hypotheses is open to confirmation bias. Such a bias is among the documented sources of the “replication crisis” plaguing many sciences at the present time and the requirement that any two agents who share the same background information is vague with respect to what counts as “the same” background information.

The EBM variant is well-adapted to a consideration of a much wider array of information, much of it indirect, than traditional OB (and, for that matter, the standard Frequentist theory), which restricts the sampling to data entailed by the particular hypothesis being evaluated. If Bradley Efron is himself taken as a model EBM statistician, this stance is open to ideas as well as information on a large and welcome scale.

Evidentialism provides a very different way of looking at the testing of hypotheses/models. Since it necessitates the comparison of two or more hypotheses/models at a time, it helps undermine confirmation bias [38,39]. In our view, it supplements Bayesianism and is not a rival to it (although the emphasis is placed on the *objectivity*-guaranteeing and not the *truth*-obtaining character of our statistical inferences).

Another way of comparing the current and emerging paradigms is with respect to the source of the unavoidable uncertainty in the statistical inferences we make, whether in the practice of science or the assessments of everyday life. As far as Basic or Subjective Bayesianism is concerned, it has to do with our ignorance concerning how things really are, an ignorance that will be remediated in the course of time. As far as what we are calling traditional Objective Bayesianism, it stems from a lack of constraint in the determination of our prior probability beliefs. On its Empirical Bayes Methods variant, the source of uncertainty is the incompleteness—indeed, the ever-growing amount—of our data and the lack of enough if not all the methods needed to analyze and order it. That said, the Objective variants share with their Subjective Bayesian precursors the view that, eventually, statistical inference will no longer be uncertain, at least as far as its scientific applications are concerned and to the extent that the same priors are eventually shared. Finally, for Evidentialism, the source of uncertainty is to be found in our intellectual finitude, which is to say our limited ability at any given time to develop new theories, new and more insightful ways of looking at the world to explain and predict the data, and, with these new theories, more adequate models to test.

## Figures and Tables

**Table 1:** Matrix summary of the fair/biased coin flip results.

	Positive	Negative
The coin is biased	0.4096	0.5904
The coin is unbiased	0.1562	0.8438

**Table 2:** Bayesian variants as a function of whether the prior is informative/non-informative and objective/subjective.

	Informative Prior	Non-Informative Prior
**Objective prior**	Empirical Bayes	Objective Bayes
**Subjective prior**	Subjective Bayes	Logical Bayes

## Data Availability

The data presented in this study are available on request from the corresponding author.
